# Rifampin as a novel trigger of drug-induced enterocolitis syndrome

**DOI:** 10.5588/ijtldopen.26.0133

**Published:** 2026-07-13

**Authors:** A. Tamborino, G. Liccioli, L. Tomei, E. Chiappini, S. Trapani, F. Mori, L. Galli, E. Venturini

**Affiliations:** 1 Pediatric Infectious Diseases Unit, Meyer Children’s Hospital IRCCS, Florence, Italy;; 2 Allergy Unit, Meyer Children’s Hospital IRCCS, Florence, Italy;; 3 Department of Health Sciences, University of Florence, Florence, Italy;; 4 Pediatric Unit, Meyer Children’s Hospital IRCCS, Florence, Italy.

**Keywords:** tuberculosis, paediatric, gastrointestinal, non-IgE-mediated drug hypersensitivity, beta-lactam antibiotics

Drug-induced enterocolitis syndrome (DIES) is a non-IgE-mediated hypersensitivity reaction primarily affecting the gastrointestinal tract. It typically occurs 1–4 h after ingestion of the culprit drug and can present with severe symptoms such as vomiting, pallor, lethargy, hypotension, and, in some cases, hypovolemic shock.^1,2^ Despite its potential clinical severity, DIES remains underrecognised worldwide. Early diagnosis and prompt management are crucial for preventing complications and improving patient outcomes.^3^ The true incidence of DIES remains undefined; a paediatric allergy unit in Italy reported an incidence of approximately 0.4%, among 714 suspected drug hypersensitivity reactions (DHRs).^2,4^ DIES has been most frequently associated with antibiotics, particularly amoxicillin and amoxicillin/clavulanate,^1–9^ with rare cases involving paracetamol and proton pump inhibitors.^10,11^

We present what appears to be the first documented case of rifampin-induced DIES in a 15-year-old girl undergoing treatment for pulmonary TB.

## CASE PRESENTATION

A previously healthy 15-year-old girl was diagnosed with active pulmonary TB associated with lymphadenitis, confirmed by clinical evaluation, chest radiography, and microbiological testing. She was started on a standard first-line anti-TB regimen consisting of rifampin, pyrazinamide, ethambutol, and isoniazid. Four days after initiation of treatment, the patient developed mild gastrointestinal symptoms, including nausea, vomiting, and abdominal pain, which progressively worsened over the subsequent days. On the fifth day of therapy, she experienced recurrent episodes of vomiting occurring a few hours after the administration of rifampin and ethambutol, accompanied by marked lethargy. On one occasion, vomiting occurred within 30 min of ingesting pyrazinamide, further suggesting a drug-related reaction. Due to the persistence and severity of vomiting, she presented to the emergency department (ED).

Laboratory tests revealed a reduced haematocrit (34.6%), elevated liver enzymes (alanine aminotransferase, ALT, 196 U/L and aspartate aminotransferase, AST 435 U/L), and a mildly increased C-reactive protein (CRP) level (2.3 mg/dL), suggesting hepatocellular injury. On clinical evaluation, capillary refill time was prolonged (3–4 s), with periorbital darkening and dry mucous membranes; the remainder of the physical examination was unremarkable. She was diagnosed with drug-induced liver injury (DILI) and dehydration. All anti-TB therapies were immediately stopped, and the patient received supportive therapy with intravenous fluids. After clinical improvement and resolution of vomiting, she was discharged from the ED with a recommendation for follow-up after 1 week.

One week later, at follow-up, blood tests showed significant improvement in liver function and normalisation of haematocrit levels. The patient was subsequently admitted for further monitoring and possible reintroduction of anti-TB therapy. After consultation with the infectious diseases team, and in accordance with established guidelines for managing hypersensitivity reactions to antitubercular agents,^12–14^ rifampin and ethambutol were reintroduced, while pyrazinamide and isoniazid were withheld. The decision to perform rechallenge was made in a monitored inpatient setting given the potential risk of severe reactions, including hypotension and shock. The patient developed vomiting, nausea, drowsiness, and lethargy approximately 2 h after rifampin administration. Her blood pressure was 105/60 mmHg. Laboratory tests revealed mildly elevated procalcitonin (2.41 ng/mL), CRP (1.49 mg/dL), slight neutrophilic leucocytosis (11,880/mm^3^, with 92.2% neutrophils), and mild liver enzyme elevations (ALT 35 U/L, AST 89 U/L). Arterial blood gas analysis showed mild metabolic alkalosis (pH 7.6, HCO_3_^−^ 26.6 mmol/L) with lactate levels of 17 mg/dL. Methaemoglobin levels were within normal limits (Figure). Based on the reproducible delayed vomiting, absence of cutaneous or respiratory signs of IgE-mediated DHRs, and systemic inflammation markers, DIES was considered the most likely diagnosis. The case met the diagnostic criteria proposed by Van Thuijl et al.^3^ which included one major criterion (vomiting occurring 1–4 h post-drug ingestion) and at least three minor criteria (lethargy, pallor, neutrophilia, and need for intravenous fluids). Other causes of delayed gastrointestinal reactions, including infectious gastroenteritis and direct drug-induced irritation, were excluded. Rifampin was permanently discontinued, and levofloxacin was introduced as an alternative in combination with isoniazid, ethambutol, and pyrazinamide. The patient tolerated the adjusted regimen well, with no recurrence of gastrointestinal or systemic symptoms, and successfully completed 6 months of anti-TB therapy. Written informed consent for data publication was obtained from the parents of enrolled children.

**Figure.**
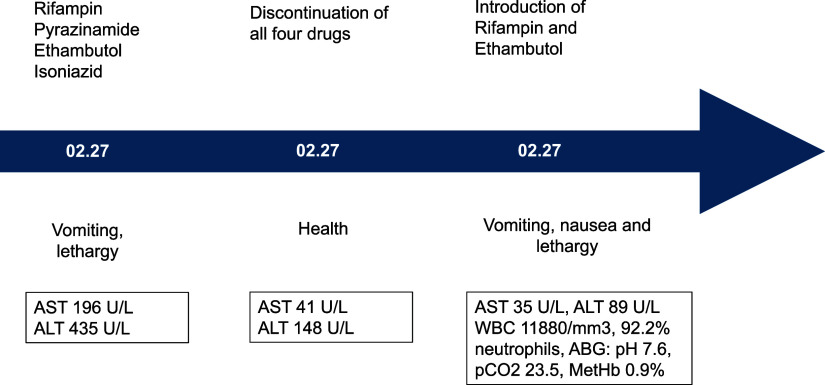
Clinical course and laboratory findings during wash-out period.

## DISCUSSION

To our knowledge, this is the first reported case of rifampin-induced DIES. This observation underscores the diagnostic challenges associated with DIES, particularly in distinguishing it from more common adverse drug reactions such as DILI or infectious gastroenteritis. In our case, the initial diagnosis of DILI was revised following the clear temporal association between rifampin intake and delayed vomiting, confirmed upon rechallenge. DIES was first described in 2014 by Novembre et al.^9^ in a 6-year-old girl who developed repetitive vomiting 1–2 h after amoxicillin ingestion. The syndrome resembles food protein–induced enterocolitis syndrome, a non-IgE-mediated food allergy presenting with similarly delayed gastrointestinal symptoms. Since then, several paediatric and adult cases have been reported, primarily in Europe, most often associated with beta-lactam antibiotics, especially amoxicillin or its combinations^1–9^; rare cases involving paracetamol and pantoprazole in adults have also been described.^2^ Recently, Özer reviewed the paediatric literature, identifying 10 patients aged 1–4 years and describing their clinical features, trigger drugs, latency between drug intake and clinical manifestations, laboratory abnormalities, and treatments.^1^

No prior reports of rifampin-induced DIES exist, making this a novel observation. Distinguishing DIES from other rifampin-associated adverse reactions is essential. Although rifampin commonly causes gastrointestinal symptoms as direct adverse effects, DIES is characterised by delayed onset (1–4 h post-ingestion), reproducibility upon rechallenge, and accompanying systemic inflammatory markers including neutrophilia and elevated acute phase reactants.^15,16^ Rifampin is, also, recognised for causing hypersensitivity reactions, including systemic hypersensitivity with fever, rash, hepatotoxicity, and severe cutaneous adverse reactions such as DRESS syndrome, Stevens–Johnson syndrome, and toxic epidermal necrolysis.^12,13^ However, the specific presentation as an enterocolitis syndrome without cutaneous manifestations has not been previously documented. The underlying immunopathology is thought to involve T-cell-mediated delayed hypersensitivity triggered by drug metabolites that damage intestinal epithelial cells or alter gut permeability.^17^ Diagnosis remains clinical, relying on the temporal relationship, symptom reproducibility, exclusion of IgE-mediated reactions, and resolution after drug withdrawal. The elevated neutrophil counts, CRP, and procalcitonin levels observed are consistent with previous DIES report.^2,3^ Metabolic acidosis and alkalosis are not pathognomonic but reflect gastrointestinal fluid losses and haemodynamic compromise; severe cases typically present with metabolic acidosis hyperchloremic or lactic, while early vomiting-dominated phases are more often associated with metabolic alkalosis.^15^ Rifampin’s immunomodulatory properties may contribute to DIES pathogenesis. Rifampin binds myeloid differentiation protein 2 (MD-2), blocking TLR4 signalling and reducing proinflammatory mediators.^18^ Additionally, rifampin activates the pregnane X receptor (PXR) in intestinal epithelial cells, constraining mucosal NF-κB activity; this crosstalk between xenobiotic metabolism and inflammation may explain aberrant gastrointestinal immune responses in susceptible individuals.^19^ Nonreaginic antirifampin antibodies detected in patients with hypersensitivity reactions suggest that rifampin or its metabolites may act as haptens, though the potential for triggering T-cell-mediated delayed hypersensitivity warrants further investigation.^19^

The management approach in this case aligns with current recommendations.^12,13^ Bermingham et al.^13^ emphasised that adverse drug reactions occur in over 60% of patients on anti-TB therapy, representing significant barriers to treatment adherence. Mutlu et al.^20^ proposed a pragmatic approach to emphasising clinical risk stratification with sequential rechallenge and rapid desensitisation when appropriate. According to American Thoracic Society/Centers for Disease Control and Prevention/Infectious Diseases Society of America (ATS/CDC/IDSA) guidelines, when severe systemic reactions occur, all potentially causative drugs should be discontinued^12^; rechallenge should be performed in a monitored setting with sequential reintroduction.^12,13^ The guidelines recommend restarting rifampin first as the most potent drug, followed by other agents.^12^ When rechallenge confirms a severe hypersensitivity reaction, permanent discontinuation is warranted.^12^ The successful substitution of levofloxacin for rifampin demonstrates that effective alternative antitubercular regimens can be implemented when DIES is identified.

## CONCLUSION

This case of rifampin-induced DIES emphasises the importance of considering this rare condition in patients presenting with delayed vomiting and systemic symptoms following drug ingestion, particularly when cutaneous and respiratory manifestations are absent. Clinicians managing TB should be aware that DIES may present as a distinct hypersensitivity reaction pattern to rifampin, differing from the more commonly recognised cutaneous and hepatic manifestations.^12,13^ Further research is needed to elucidate its pathophysiology, identify biomarkers, and develop standardised diagnostic criteria.
